# Association of Competitive Adolescent Athletes from Lean and Non-Lean Sports Physical, Social and Psychological Characteristics with Adherence to Mediterranean Diet

**DOI:** 10.3390/sports12100267

**Published:** 2024-09-29

**Authors:** Fani Thoma, Eirini Koidou, Christina Dolopikou, Vassilis Barkoukis, Constantinos Giaginis, Sousana K. Papadopoulou

**Affiliations:** 1 Schools of Physical Education and Sports Science (Serres), Faculty of Physical Education and Sport Sciences, Aristotle University of Thessaloniki, 621 22 Serres, Greece; fanithoma@phed-sr.auth.gr (F.T.); or rkoidou@gmail.com (E.K.); cdolopik@phed-sr.auth.gr (C.D.); 2 Department of Nutritional Sciences & Dietetics, Faculty of Health Sciences, International Hellenic University, Sindos, 570 01 Thessaloniki, Greece; 3 Department of Physician Education and Sport Sciences, Aristotle University of Thessaloniki, Thermi, 541 24 Thessaloniki, Greece; bark@phed.auth.gr; 4 Department of Food Science and Nutrition, School of Environment, University of Aegean, 814 00 Myrina, Greece; cgiaginis@aegean.gr

**Keywords:** adolescent athletes, lean and non-lean sports, Mediterranean diet, theory of planned behavior

## Abstract

Mediterranean diet (MD) is regarded as one of the healthier dietary patterns which is recommended for athletes. This study aims to investigate the adherence to the Mediterranean diet (AMD) and associated beliefs in a large, diverse sample of competitive adolescent athletes from various sports, including both lean and non-lean sports. Additionally, the study examines factors important regarding intention to AMD within the Theory of Planned Behavior (TPB). In the study took part 711 adolescents (357 male, aged = 14.93 ± 1.38, and 354 female, aged = 14.85 ± 1.35) athletes. The level of AMD was not a significant different between lean (mean = 4.98, SD = ±3.90) and non-lean (mean = 4.77, SD = ±3.68) sport. According to the sport type the 35% of lean sport athletes demonstrated low AMD, 34.1% moderate adherence and 30.9% a high degree. The non lean athletes demonstrated low AMD in 36.3%, moderate adherence 33% and high degree in 30.7%. The results of the mediation analysis indicated a significant mediation effect of intention in the relationship between TPB variables and MD. Based on the results of the study the type of sport does not play a role in the AMD, which, as in the general population, is low. Also validate the TPB and underscore the significance of targeting individuals’ intentions to promote positive dietary behaviors.

## 1. Introduction

A healthy diet and beneficial eating habits are vital and important components of healthy lifestyles in all life stages [1,2]. However, a suitable diet becomes significantly important during adolescence due to it being the second most rapid period of growth in life, behind infancy. Research indicates that maintaining a suitable diet is crucial for promoting optimal physical, skeletal, and cognitive growth, mitigating weight-related problems, and decreasing the likelihood of sickness [3,4]. Inadequate nutritional intake can have severe consequences across/throughout the reproductive years and beyond [5]. Adequate diet and optimal nutritional state are of utmost importance for a teenage athlete. Various global organizations and research highlight the correlation between athletic performance and the process of recuperating from physical activity, which is enhanced by consuming the most suitable nourishment [6,7,8,9]. 

Athletic performance in sports requires a well-balanced diet to reduce fatigue and minimize the chances of diseases and injuries. Additionally, it allows athletes to enhance their training and expedite the recovery process. Energy balance is crucial for facilitating recovery from exercise stress and maximizing training adaptations. Despite the existence of dietary recommendations for young athletes, which are designed to assist them in meeting their heightened nutrient needs, these recommendations are not consistently followed by the athletes [10,11,12,13]. At the present time, there is a scarcity of research examining dietary habits among competitive athletes across various sports. The majority of these studies focused on examining the consumption of macronutrients and micronutrients, as well as hydration and weight management [14,15]. In 2019, Νoll et al. [16], asserted that there was a correlation between the type of sport and the amount of time spent on practice, and how it affected the eating habits. Experienced athletes exhibit a higher frequency of consuming nutritious food over an extended period of time. In a subsequent study [17] the authors discovered that sociodemographic parameters, body weight control, and sedentary factors significantly influence the eating behaviors of high school athletes. Iglesias-Gutiérrez et al. [18], found differences in soccer players aged 16–21 years old on food choice patterns in relation to the playing position. Carcía-Rovés et al. [19], notice that male and female soccer players do not follow a dietary pattern both before and on the day and the day after the match. Juzwiak [20] (2021) advocated that the primary factors influencing the eating habits of sportsmen in terms of aesthetics and weight class are the pursuit of an idealized physique and the culinary traditions of their home country. 

### 1.1. Mediterranean Diet Health Benefits and Athletes

The Mediterranean Diet (MD) is widely studied in general population and is one of best eating pattern for the prevention of cardiovascular diseases, reduces inflammatory markers, improves blood vessel function, reduces the risk of metabolic syndrome and diabetes, is associated with cancer prevention, improves mental health, increases lifespan and is environmentally friendly [21]. Importantly, the United Nations Educational, Scientific and Cultural Organization (UNESCO) considers the Mediterranean diet an “Intangible Cultural Heritage of Urgent Safeguarding” [22]. The MD includes proportionally high consumption of untreated cereals and whole grains, legumes, fruits and vegetables, nuts, seeds, herbs and spices, extra virgin olive oil and moderate amounts of meat products, poultry, fish, seafood, dairy, eggs, and sweets. Meals are planned around these foods and are low in saturated fat with high amounts of monounsaturated fat and fiber. Consequently, MD includes high amounts of antioxidants, fiber, complex/low-glycemic index carbohydrates and low saturated fatty acids. It’s also rich in vegetable proteins and has a balanced ratio of n-6/n-3 fatty acids [23,24]. Also, breakfast and family meals are a part of the Mediterranean diet.

High adherence to MD in children and adolescents has been linked with a lower prevalence of overweight and obesity [25,26], increased cognitive performance [27,28,29], and health related quality of life [30,31]. Also, MD is compliant with nutritional recommendations for sport performance [32,33,34]. Nevertheless, numerous studies have documented a notable decrease in adherence to the Mediterranean diet (MD) among those who engage in athletic activities. To be more precise, several studies utilizing the KIDMED method, with a limited number of participants and across several sports, have demonstrated a moderate level of adherence to the Mediterranean diet. Furthermore, these studies have identified variances in adherence between male and female athletes, as well as variations between different sports [26,32,34,35,36]. Most research emphasize the significance of MD and the need to identify the elements that can potentially enhance adherence in order to create successful intervention programs. 

### 1.2. Theory of Planned Behavior and Eating Habits

Changing a habit or practice is a complex task. When it comes to eating habits, it is widely recognized that changing them can be more challenging than just reducing the consumption of specific meals and increasing the intake of healthier alternatives [37]. Planned behavior theory (TPB) is among the most prominent theories in understanding human behavior [38]. TPB holds that intention drives human conduct. Attitudes, subjective norms, and perceived behavioral control affect this purpose. Convictions about the probable consequences of behavior and subjective assessments of those consequences as favorable or negative influence behavior. Positive attitudes about a behavior increase the likelihood of expressing a stronger intention to engage in it. Subjective norms are the perceived social pressure or influence from family, friends, and coworkers on a behavior. This includes societal expectations and an individual’s drive to meet them. Subjective standards of significant strength might affect a person’s behavior intention positively or negatively. Finally, perceived behavioral control (PBC) is a person’s subjective appraisal of their behavior execution ability. These elements influence the individual’s intention to undertake the behavior. serves as a robust indicator of subsequent behavior. The strength of an individual’s intention to engage in a behavior predicts their likelihood of doing so [39]. The Theory of Planned Behavior (TPB) has been widely applied in various fields, including sports, physical activity, and health psychology, to better understand and predict human actions and behaviors. By recognizing and understanding the different factors that impact people’s intentions, researchers and practitioners can develop and apply strategies that effectively encourage desired behaviors and foster positive changes in individuals and communities. 

### 1.3. Study Importance and Novelty

While adolescent athletes acknowledge the importance of nutrition for athletic performance, this understanding does not always result in the adoption of suitable dietary practices. [40,41]. Thus, it is crucial to understand the factors that contribute to the adoption of MD. TPB may be an effective framework to predict and identify young athletes’ diet and eating behaviors. In their review, Riebl et al. [42], supported those attitudes had the strongest association with intentions towards dietary behaviors. Berg et al. [43] indicated that knowledge about nutrition predicted dietary behaviors of oldest children and girls through their attitudes. Morissette et al. [44] found strong correlation between past behavior and intention to use restrictive dietary behaviors to lose weight and no differences between athletes and general population Attitudes and past behavior were also significant predictors of intention to adopt dietary behaviors in the Laramée et al. [45] intervention. Furthermore, perceived parental support was found important in modeling the healthy eating habits in normal weight adolescent male and female soccer players in a two-year obesity prevention Intervention program [46].

### 1.4. Study Aims and Rationale

Existing evidence largely focuses on examining sport nutrition knowledge, food choices, and improving restrictive dietary behaviors, whereas the studies using TPB have small samples from a limited number of sports. There is need to investigate the factors that predict the adherence to MD and lifestyle, and the related beliefs in a large sample of competitive athletes from different sport (lean and non-lean). Therefore, the aim of this study was to evaluate the adherence to MD in competitive adolescents’ athletes between lean and non-lean sports. In addition, the present study seeks to assess factors important to competitive young athletes regarding intention to adherence to MD within the TPB.

## 2. Materials and Methods

### 2.1. Ethics

Ethical approval was obtained from the Bioethics Committee of Aristotle University of Thessaloniki, specifically the Ethics Committee of the School of Physical Education and Sport Sciences (Serres), under protocol number ERC-014/2022.

### 2.2. Data Collection

This cross-sectional study was conducted as a component of a project that examines the correlation between coaches’ nutritional expertise and the dietary habits of their athletes. The study recruited all athletes who participated through internet publicity (advertisements) and personal communication from the authors. Prior notification was given to coaches, parents, and athletes of the purpose of the study. The athletes’ parents were requested to provide written consent. The consent form outlined the objective of the study and the process for filling out the questions. Additionally, the survey leader’s contact information was supplied. Parents were requested to indicate if their child had a diet/nutrition-related ailment (such as eating disorders, diabetes, celiac disease) or if their child adhered to a plant-based diet or a specific diet (such as weight reduction). In these instances, the athlete was excluded from the study. Subsequently, with the coach’s authorization, the researchers proceeded to inspect the sports facilities and, upon verifying the signed consent forms, conducted anthropometric evaluations (including weight, height, and body mass index). Additionally, the athletes anonymously completed the questionnaires. Each athlete underwent individual evaluation during a training session. The data is gathered from professionals.

### 2.3. Participants 

A total of 711 adolescents (357 male, aged = 14.93 ± 1.38, and 354 female, aged = 14.85 ± 1.35) athletes participated in the study. The athletes divided into two groups, lean and non-lean sports. The lean category includes aesthetic, weight-dependent and endurance sports (n = 233). The non lean category includes ball game, power, and technical sports (n = 488). All athletes were competitive with at least 2 years of systematic training, and they had participated in national and international competitions or international meeting. At this age many children, in Greece, participate in more than one sport, the study included athletes training in only one sport.

### 2.4. Measures

#### 2.4.1. Demographic Characteristics

Data were collected using a self-report questionnaire. Athletes were asked to self-report their sex, date of birth, years practicing their sport, frequency and duration of training, participation in national and international competitions, and sporting awards. The questionnaire included questions related to their training experience, they also recorded their eating habits before during and after training and whether they had consulted a dietician. In addition, they were asked whether coach gave them nutritional advice, for example frequency, and whether he/she weighed them.

#### 2.4.2. Anthropometric Characteristics

An anthropometric assessment was carried out by an expert. Body weight measured with a portable scale (Seca 804 Sensa). Height (m) was measured with a portable stadiometer (Seca 214, Hamburg, Germany). Both measurements were performed twice, and averages were used. Participants were weighed with light clothes and without shoes. Body mass index (BMI) was calculated for each participant as body weight (kg), divided by height (m^2^). The BMI was categorized according to WHO—growth chart according to age for the classification of weight status as underweight, normal weight, overweight or obese [47]. 

#### 2.4.3. Theory of Planned Behavior 

The constructs of the TPB, were evaluated according to guidelines described by Ajzen [48]. The questionnaire included four components, attitudes, subjective norms, perceived behavioral control and intention. The questionnaire commenced with a concise explanation of the MD, upon which the respondents based their answers. These items were adapted from previous research [49,50,51]. The focus of our investigation was the ‘adherence to the Mediterranean diet to increase athletic performance’.

#### 2.4.4. Attitudes towards Mediterranean Diet

Attitudes were assessed by the stem question ‘Adopting Mediterranean diet to increase my athletic performance is…’. Participants responded on 5 bipolar adjectives (i.e., harmful-beneficial, unpleasant-pleasant, worthless-useful, unhealthy-healthy and unimportant-important) in a 7-point scale (1 = negative pole, 7 positive pole). The mean score was used to measure attitudes with the higher the score, the more positive the attitudes. 

#### 2.4.5. Perceived Behavioral Control

Perceived control was assessed by the mean of four (e.g., ‘If I wanted to, I could follow a MD diet program for the next 6 months’). Participants responded on a 7-point Likert-type scale ranging from 1 (very difficult/strongly disagree) to 7 (very easy/strongly agree). Higher scores indicated greater perceived behavioral control. 

#### 2.4.6. Subjective Norm

Subjective norm was measured from the mean of four items (e.g., ‘Most people important to me would approve me having a MD diet program in the next 6 months’). Responses were anchored on a 7-point Likert-type scale ranging from 1 (extremely unlikely/strongly disagree) to 7 (extremely likely/strongly agree). Higher scores indicated higher subjective norm.

#### 2.4.7. Intention

Intentions were calculated from the mean of three questions (e.g., ‘I intent to follow a MD diet program in the next 6 months’). Responses were recorded on a 7-point Likert-type scale ranging from 1 (extremely unlikely/strongly disagree) to 7 (extremely likely/strongly agree). Higher scores indicating stronger intention to AMD.

### 2.5. Assessment of Mediterranean Diet Adherence

The Mediterranean Diet Quality Index (KIDMED) was used to evaluate the MD. The index was developed for kids and adolescents and consists of 16 items which evaluate the frequency of consumption of food groups. The index ranges from 0 to 12. Four questions related to negative habits to the MD are assigned a value of –1, and those (12 questions) with a positive aspect are assigned a value of +1. The sums of the values range between 0 to 12. The sums of the values were classified into three levels: score ≥ 8 is considered “optimal MD”, 4–7 as “average MD adherence, improvement needed to adjust intake to Mediterranean patterns”, and ≤3 as “very low diet quality” [52].

### 2.6. Data Analyses

The Statistical Package for the Social Sciences (SPSS), version 28, was used to analyze the data. The distribution of continuous variables was checked for normality before further analysis. The Kolmogorov–Smirnov Test was used to check the normality of the variables, followed by Levene’s test to determine the homogeneity (*p* > 0.05). Cronbach’s alpha values were calculated to assess the internal consistency of the items. The independent samples *t*-test was used to examine differences between lean and non-lean sports in the mean scores of ages, height, weight, BMI and KIDMED scores. Cohen’s d was calculated to assess the effect size of differences. For the categorical variables a Chi-squared test was applied with the Fisher’s exact, when necessary. The Pearson’s r correlation coefficient was used for investigating the relationships between total KIDMED score and the TPB variables. 

Following this, the PROCESS macro was utilized to conduct a regression-based path analysis, a methodology akin to structural equation modeling but accounting for non-normal sampling distributions [53]. We employed a mediation analysis to investigate whether the relationship between TPB variables and adherence to the Mediterranean diet was mediated by intentions. The primary objective of this model was to analyze the total (path c) and direct effects (paths a, b, c’), which represent the unstandardized regression coefficient and significance between the independent and dependent variables in each respective model. The present study also examined the indirect effect (IE) which signifies the alteration in MD for each unit modification in TPB variables that are mediated by intentions. The mediation hypotheses were tested using the bootstrapping methods recommended by Preacher and Hayes (2008) [54]. A resampling technique involving the generation of 5000 bootstrap samples was employed. The study calculated point estimates and confidence intervals at a 95% level for the indirect effect. Significance was determined by whether the confidence interval excluded zero. The statistical analyses were conducted using IBM SPSS Statistics for Windows version 28. A significance level of *p* < 0.05 was established for all analyses.

## 3. Results

### 3.1. Descriptive Analysis

All variables had a normal distribution (*p* > 0.05). Cronbach’s alphas were >0.7 for all subscales. The age, height, weight, and BMI of the participating male and female athletes and according to their type of sport, are presented in Table 1. No differences were pointed between the different type of sport, concerned their age (t_(709)_ = −0.304, *p* = 0.762), height (t_(709)_ = −0.724, *p* = 0.470), wight (t_(709)_ = −0.491, *p* = 0.312) and BMI (t_(709)_ = 1.872, *p* = 0.062). Chi-square test of independence applied for the assessment of differences between the type of sport and BMI categories. No differences were pointed between the different type of sport.

The competition frequency and weekly training frequency of the participating athletes according to their type of sport, are presented in Table 2. Chi-square test of independence applied for the assessment of differences between the type of sport. Differences were pointed between the different type of sport concerned the race frequency χ^2^(2, Ν = 711) = 82.29, *p* < 0.001 and concerned the weekly training frequency χ^2^(2, Ν = 711) = 25.527, *p* < 0.001. 

Τhe results of the Chi-square test of independence to assess differences between the type of sport and the participants’ sources of nutritional information are shown in Table 3.

Τhe results of the Chi-square test of independence to assess differences between the type of sport and coach’s behavior are shown in Table 4.

### 3.2. Level of MD Adherence

The mean score of MD was 4.83 (SD ± 3.75) ranged from 0–12 points for all the participants. According to the KIDMED index, 35.9% of the athletes showed a low adherence of MD, 33.3% moderate adherence and 30.8% a high degree. According to the sport type the 35% of lean sport athletes demonstrated low adherence of MD, 34.1% moderate adherence and 30.9% a high degree. The non lean athletes demonstrated low adherence of MD in 36.3%, moderate adherence 33% and high degree in 30.7%.

The level of adherence to the MD was not a significant different in the score between lean (M = 4.98, SD = 3.90) and non-lean (M = 4.77, SD = 3.68) sport; t _(709)_ = 0.716, *p* = 0.474, Cohen’s = 3.753, CI: −0.378 to 0.813. 

### 3.3. Correlations between TPB Variables, and KIDMED Total Score for Each Group of Participants

The Pearson correlations among TPB variables, and KIDMED for lean sport in Table 5 and for non-lean sport in Table 6. The results showed significant positive correlations between KIDMED scores and each TPB variables.

The differences between the lean and non-lean sports in the measured variables are reported in Table 7. Significant differences were found on PBC and subjective norms.

### 3.4. Effect of TPB Variables on Mediterranean Diet

The results of the mediation analysis using the PROCESS macro by Hayes [53] indicated a significant mediation effect of intention in the relationship between TPB variables and Mediterranean diet. The total effect of attitudes, subjective norms and PBC on Mediterranean diet was found to be β = 0.94, *p* < 0.001, β = 0.53, *p* < 0.001 and β = 0.60, *p*< 0.001 respectively. The direct effect of attitudes, subjective norms and PBC on mediterranean diet, after controlling for intentions, was β = 0.30, *p* < 0.05, β = 0.10, *p* < 0.05 and β = 0.19, *p* < 0.05 respectively. In all cases, the direct effect was non-significant (*p* > 0.05). The indirect effect of attitudes, subjective norms and PBC on mediterranean diet through intentions was β = 0.63, *p* < 0.001, β = 0.43, *p* < 0.001 and β = 0.41, *p* < 0.001 respectively, and was significant in all cases. The non-significant direct effects and the significant indirect effects indicate full mediation of intentions to the effect of attitudes, subjective norms and PBC on mediterranean diet (Table 8).

## 4. Discussion

Various studies have repeatedly demonstrated that athletes, particularly those engaged in sports that place emphasis on weight and appearance, have a greater incidence of unhealthy or unsustained eating habits. This study assessed the adherence to the Mediterranean Diet (AMD) in a substantial group of adolescent competitive athletes participating in various sports, including both lean and non-lean sports [55]. The primary results indicated that around 30.9% of lean sport athletes and 30.7% of non-lean sport athletes exhibited a significant level of adherence to the Mediterranean diet. Curiously, almost 30% of athletes reported having a low AMD. Our findings are consistent with previous research conducted on adolescents of similar age [56,57]. These data suggest that adolescent competitive athletes had similar results and a modest level to AMD compared to the general adolescent population. Moreover, our research uncovered that athletes participating in sports that require a slim physique did not exhibit a higher prevalence of age-related macular degeneration (AMD) compared to athletes in sports that do not prioritize leanness. No studies have been found that examine potential disparities in age-related macular degeneration (AMD) between physically fit and non-physically fit sports among adolescent athletes competing at a high level. These data indicate that a significant number of adolescent athletes may not be following the MD properly, regardless of whether they participate in lean or non-lean sports. The intense focus on athletics at a competitive level does not appear to have acted as a catalyst for the AMD. Regarding the absence of notable distinctions between lean sport athletes and non-lean athletes, numerous factors can account for this outcome, such as the homogeneity of the athletes involved in the study. Athletes, regardless of the specific sport they participate in, may share similar nutritional practices, eating habits, or preferences. In their study, Calella et al. [32] found that athletes participating in volleyball, long-distance swimming, and gymnastics consistently had suboptimal AMD levels. They also observed a weak positive correlation between dietary awareness and AMD in all individuals. 

Our study involved soliciting players to indicate whether they have consulted a sports nutritionist for guidance on sports nutrition. Out of all the participants, 33.3% responded negatively. There was no notable distinction observed between individuals involved in lean sports and those involved in non-lean sports (Cramer’s V = 0.004). However, we observed notable disparities in responses to the question “Did your coach provide you with nutritional guidance?” The value of Cramer’s V for the question “how often does the coach weigh you?” is 0.20, while for the other question it is 0.23. A study found that physically fit players were more inclined to follow their coaches’ suggestions and undergo frequent weigh-ins [58]. This discovery prompts worries regarding the influence of coaches on the dietary habits of athletes. The involvement of coaches in directing players’ dietary decisions is a notable issue, necessitating the implementation of structured teaching initiatives led by formal sports organizations. Moreover, extraneous variables such as athletic culture, daily routines, school hours, availability to specific meals, socioeconomic reasons, or familial influences could have similarly influenced both groups. Philippou [34] demonstrated that implementing an intervention that included activities for parents resulted in a significant improvement in adherence to the Mediterranean diet among the participants. This highlights the potential beneficial impact of targeted nutritional education programs that actively include both parents and coaches. 

Furthermore, although the fundamental nutritional principles are applicable to all sports, the absence of discrepancies may be attributed to the distinct nutritional demands of each sport and some sport-specific factors. Endurance or team athletes may primarily emphasize carbohydrate loading tactics, whereas strength or weight-dependent athletes may prioritize protein consumption to facilitate muscle recovery, growth, or weight loss. Nevertheless, these distinctions are frequently nuanced and tailored to the particular requirements of the sport, rather than being determined by whether it is categorized as a lean or non-lean sport. When examining disparities between lean and non-thin athletes, it is crucial to take into account aspects that go beyond simply following a particular dietary regimen such as the Mediterranean diet. 

Due to the multifactorial nature of AMD, we analyzed the training and anthropometric features of the groups. There were notable variations in the weekly training frequency and race frequency between the groups, which were influenced by the type of sport. Sports that are not focused on lean body types had a higher number of training days per week (Cramer’s V = 0.18) and a higher number of races per year (Cramer’s V = 0.34). This condition likely accounts for the increased number of training days per week, although it may not necessarily have a direct impact on individuals’ dietary choices. Noll et al. [16], on the other hand, discovered that the types of sports, specifically team sports, and the amount of time spent training were linked to eating habits. Their survey assessed healthy eating as “having lunch and dinner with parents” and identified unhealthy eating habits as “eating in front of the TV.” It also measured the frequency of consuming both good and unhealthy food items in the past 7 days. The results did not appear to vary based on the type of sport while using the KIDMED application. 

Most of the sportsmen had a body weight within the normal range when considering anthropometric measurements. No distinctions were identified among the various types of sports in terms of BMI and BMI categories. The intense focus on sports at a competitive level appears to function as a protective factor against weight-related problems in the general population. Our findings are consistent with other studies that included adolescents of a similar age [56,57]. Prior research in this field has demonstrated associations between adherence to the Mediterranean diet, body weight, and body mass index (BMI). In a study conducted by Natour on Palestinian football players, it was discovered that there is a correlation between the amount of AMD and both performance and weight status [59]. Alacid et al. [60] discovered comparable results in female canoeing athletes, while Marninez-Rondriguez [61] found similar results in beach handball players from Spain. However, Santata et al. [42] discovered that a considerable proportion of female adolescent rythmic gymnastics athletes had a low AMD. Nevertheless, no significant correlation was seen between AMD and anthropometric factors.

With respect to the theory of planned behavior variables, our study demonstrated that attitudes, subjective norms, and perceived behavioral control (PBC) significantly predicted athletes’ intentions to adopt the Mediterranean diet. Notably, the mediation analyses revealed that intentions fully mediated the relationship between attitudes, subjective norms, PBC, and actual adoption of the Mediterranean diet. These results are in line with prior research [62,63] supporting the idea that intentions play a crucial role in translating positive attitudes and social pressures into actual dietary behaviors. The full mediation effect underscores the significance of intentions as the mechanism through which attitudes, subjective norms, and PBC influence individuals’ dietary choices. These findings contribute to the existing literature on the TPB and health behavior [64] and emphasize the importance of fostering positive intentions to promote adherence to the Mediterranean diet among the target population. 

Furthermore, this full mediation effect has theoretical implications for understanding the mechanisms through which psychological factors impact health-related behaviors. It highlights the importance of considering behavioral intentions as a critical link between cognitive determinants (attitudes, subjective norms, and PBC) and actual behavior. By focusing on intentions, researchers and theorists can better comprehend the process by which attitudes, and social influences translate into real actions. This reinforces the relevance and applicability of the TPB framework in promoting health behaviors and adds to the growing body of evidence supporting the theory’s utility in various contexts. The findings validate the Theory of Planned Behavior and underscore the significance of targeting individuals’ intentions to promote positive dietary behaviors. By understanding the mediating process, researchers and practitioners can develop more effective strategies to encourage individuals to adopt the Mediterranean diet and, potentially, other health-promoting behaviors in diverse populations. Health interventions and public health campaigns aimed at encouraging the Mediterranean diet adoption should focus on shaping favorable intentions among individuals by addressing their attitudes, perceived norms, and perceived behavioral control in relation to this dietary pattern. By doing so, practitioners and policymakers can effectively facilitate the transition from intentions to actual behavior, leading to improved health outcomes and overall well-being.

In conclusion, this study highlights the dietary challenges faced by adolescent competitive athletes, particularly those in lean and non-lean sports, underlining common dietary practices across sport types. Despite similar AMD diet across lean and non-lean sports, a substantial portion of athletes displayed poor AMD. The findings suggest that competitive sports involvement does not significantly influence dietary adherence to the Mediterranean diet, with external factors such as nutritional education and coaching playing crucial roles. The study emphasizes the need for targeted nutritional education programs involving coaches and parents to improve dietary behaviors and overall health outcomes among young athletes. Furthermore, the study emphasizes the importance of targeting intentions in health interventions to adopt positive dietary behaviors. By addressing attitudes, perceived norms, and perceived behavioral control, experts can effectively encourage the adoption of the Mediterranean diet, leading to improved health outcomes and overall well-being. These results validate the Theory of Planned Behavior and its utility in promoting health behaviors, providing valuable insights for future research and public health strategies.

## Figures and Tables

**Table 1:** Anthropometric Characteristics.

								Confidence Interval (95%)	
	Lean (n = 223)	Non-Lean (n = 488)	Total (n = 711)	t	*p*	χ^2^ (n = 711)	Cohen’s	Lower	Upper
**Age (y)**	14.87 ± 1.37	14.90 ± 1.37	14.89 ± 1.37	−0.304	0.762		1.374	−0.2518	0.1844
**Height (m)**	1.68 ± 0.08	2.05 ± 7.80	1.93 ± 6.47	−0.716	0.470		6.464	−1.4040	0.6477
**Weight (kg)**	60.7 ± 13.68	61.22 ± 11.91	61.07 ± 12.49	−0.491	0.312		12.495	−2.4786	1.4872
**BMI (kg/h^2^)**	21.36 ± 3.54	20.85 ± 2.84	21.01 ± 3.09	1.820	0.062		3.084	0.9947	1.0370
Underweight	1.8% (n = 4)	2.3% (n = 10)				7.024 (3), *p* = 0.071			
Normal Weight	80.7% (n = 177)	86.3% (n = 415)							
Overweight	13% (n = 29)	9.8% (n = 48)							
Obese	4.5% (n = 10)	1.6% (n = 8)							

**Table 2:** Training characteristics.

	Lean (n = 223)	Non-Lean (n = 488)	Total (n = 711)	χ^2^ (n = 711)
**Race frequency**				
One time/year	57.6%	42.4%	59	82.29 (2), *p* < 0.001
2–3 times/year	65.2%	34.8%	89	
>4 times/year	23.4%	76.7%	583	
**Weekly training frequency**				
2–3 times/week	33.8%	66.3%	160	25.527 (2), *p* < 0.001
4–5 times/week	24.9%	75.1%	410	
6–7 times/week	44.2%	52.5%	141	

**Table 3:** Nutritional information.

	Lean (n = 223)	Non-Lean (n = 488)	Total (n = 711)	χ^2^ (n = 711)
**Visited dietitian**				
Yes	31.6%	68.4%	237	0.013 (1), *p* > 0.05
No	31.2%	68.8%	474	
**Nutritional advises**				
Yes	41.6%	58.4%	373	37.852 (1), *p* < 0.001
No	20.1%	79.9%	338	

**Table 4:** Coach’s advises.

	Lean (n = 223)	Non-Lean (n = 488)	Total (n = 711)	χ^2^ (n = 711)
**Never**	20.7%	79.3%	29	30.513 (5), *p* < 0.001
**1 time/month**	22.8%	77.2%	333	
**1 time/week**	38.7%	61.3%	235	
**3–4 times/week**	38.9%	61.1%	54	
**Every day**	51.9%	48.1%	52	
**More than 1 time/day** **6–7 times/week**	25%	75%	8	

**Table 5:** Pearson correlations coefficients among measured variables (n = 223).

	M	SD	1	2	3	4	5
**1. KIDMED score**	4.98	3.90	-				
**2. Attitudes**	4.04	0.58	0.14 *	-			
**3. Perceived behavioral control**	5.27	1.37	0.15 *	0.41 **	-		
**4. Subjective norms**	4.71	1.33	0.19 **	0.13	0.12	-	
**5. Intention**	4.84	1.70	0.36 **	0.35 **	0.50 **	0.39 **	-

M = mean; SD = standard deviation; * *p* < 0.05; ** *p* < 0.01.

**Table 6:** Pearson correlations coefficients (r) among measured variables (n = 488).

	M	SD	1	2	3	4	5
**1. KIDMED score**	4.77	3.68	-				
**2. Attitudes**	4.01	0.59	0.16 *	-			
**3. Perceived behavioral control**	5.51	1.23	0.19 **	0.33 **	-		
**4. Subjective norms**	5.51	1.23	0.23 **	0.28 **	0.27 **	-	
**5. Intention**	4.7	1.57	0.31 **	0.35 **	0.46 **	0.53 **	-

M = mean; SD = standard deviation; * *p* < 0.05; ** *p* < 0.01.

**Table 7:** Differences between groups on attitudes, perceived behavioral control, social norms, and intention.

	Lean (n = 223)		Non-Lean (n = 488)						Confidence Interval (95%)	
	M	SD	M	SD	df	t	*p*	Cohen’s d	Lower	Upper
**Attitudes**	4.04	0.58	4.01	0.59	709	0.499	0.618	0.59	−0.7032	0.11830
**Perceived behavioral control**	5.27	1.37	5.51	1.23	709	−2.311	0.021	1.28	−0.4431	−0.0360
**Subjective norms**	4.71	1.33	4.51	1.23	709	1.975	0.049	1.22	0.0009	0.4108
**Intention**	4.84	1.70	4.7	1.57	709	1.014	0.311	1.61	−0.1279	to 0.4004

M = mean; SD = standard deviation.

**Table 8:** Mediating effect of intentions.

	Relationship	Total Effect	Indirect Effect	Confidence Interval (95%)		t-Statistic
				**Lower bound**	**Upper bound**	
**Attitudes-** **Intentions-MD**	0.94 (000)	0.30 (0.196)	0.63	0.4353	0.8688	4.03
**Subjective norms-Intentions-MD**	0.53 (.001)	0.10 (0.408)	0.43	0.3153	0.5596	4.73
**PBC-** **Intentions-MD**	0.60 (.001)	0.19 (0.111)	0.41	0.2910	0.5487	5.65

MD = Mediterranean Diet; M = mean; SD = standard deviation.

## Data Availability

Researcher who wish to provide data can contact Professor Koidou and Ph.D. candidate Thoma Fani due to privacy restrictions.

## References

[1] World Health Organization https://www.who.int/news-room/fact-sheets/detail/healthy-diet.

[2] Childhood Nutrition Facts https://www.cdc.gov/healthyschools/nutrition/facts.htm.

[3] Norris S.A., Frongillo E.A., Black M.M., Dong Y., Fall C., Lampl M., Liese A.D., Naguib M., Prentice A., Rochat T. (2022). Nutrition in adolescent growth and development. Lancet.

[4] Das J.K., Lassi Z.S., Hoodbhoy Z., Salam R.A. (2018). Nutrition for the Next Generation: Older Children and Adolescents. Ann. Nutr. Metab..

[5] Saavedra J.M., Prentice A.M. (2023). Nutrition in school-age children: A rationale for revisiting priorities. Nutr. Rev..

[6] Bergeron M.F., Mountjoy M., Armstrong N., Chia M., Côté J., Emery C.A., Faigenbaum A., Hall G., Kriemler S., Léglise M. (2015). International Olympic Committee consensus statement on youth athletic development. Br. J. Sports Med..

[7] Kerksick C.M., Wilborn C.D., Roberts M.D., Smith-Ryan A., Kleiner S.M., Jäger R., Collins R., Cooke M., Davis J.N., Galvan E. (2018). ISSN exercise & sports nutrition review update: Research & recommendations. J. Int. Soc. Sports Nutr..

[8] Thomas D.T., Erdman K.A., Burke L.M. (2016). Position of the Academy of Nutrition and Dietetics, Dietitians of Canada, and the American College of Sports Medicine: Nutrition and Athletic Performance. J. Acad. Nutr. Diet..

[9] Burke L.M., Castell L.M., Casa D.J., Close G.L., Costa R.J.S., Desbrow B., Halson S.L., Lis D.M., Melin A.K., Peeling P. (2019). International Association of Athletics Federations Consensus Statement 2019: Nutrition for Athletics. Int. J. Sport Nutr. Exerc. Metab..

[10] Purcell L.K., Canadian Paediatric Society, Paediatric Sports and Exercise Medicine Section (2013). Sport nutrition for young athletes. Paediatr. Child Health.

[11] Smith J.W., Holmes M.E., McAllister M.J. (2015). Nutritional Considerations for Performance in Young Athletes. J. Sports Med..

[12] Berg E.K. (2019). Performance Nutrition for the Adolescent Athlete: A Realistic Approach. Clin. J. Sport Med. Off. J. Can. Acad. Sport Med..

[13] Kontele I., Vassilakou T. (2021). Nutritional Risks among Adolescent Athletes with Disordered Eating. Children.

[14] North M., Kelly A.L., Ranchordas M.K., Cole M. (2022). Nutritional considerations in high performance youth soccer: A systematic review. J. Sci. Sport Exerc..

[15] Cesanelli L., Ylaitė B., Messina G., Zangla D., Cataldi S., Palma A., Iovane A. (2021). The Impact of Fluid Loss and Carbohydrate Consumption during Exercise, on Young Cyclists’ Fatigue Perception in Relation to Training Load Level. Int. J. Environ. Res. Public Health.

[16] Noll M., Rodrigues A.P., Silveira E.A. (2019). Sport types and time spent playing sport are associated with eating pattern among Young Brazilian athletes. Asian J. Sports Med..

[17] Noll M., Rodrigues A.P.S., Silveira E.A. (2020). The health-related determinants of eating pattern of high school athletes in Goiás, Brazil. Arch. Public Health Arch. Belg. De Sante Publique.

[18] Iglesias-Gutiérrez E., García A., García-Zapico P., Pérez-Landaluce J., Patterson A.M., García-Rovés P.M. (2012). Is there a relationship between the playing position of soccer players and their food and macronutrient intake?. Appl. Physiol. Nutr. Metab..

[19] García-Rovés P.M., García-Zapico P., Patterson A.M., Iglesias-Gutiérrez E. (2014). Nutrient intake and food habits of soccer players: Analyzing the correlates of eating practice. Nutrients.

[20] Juzwiak C.R. (2021). Understanding food choices and eating practices of Brazilian and Spanish athletes in aesthetics and weight class sports. Mot. Rev. De Educ. Física.

[21] Guasch-Ferré M., Willett W.C. (2021). The Mediterranean diet and health: A comprehensive overview. J. Intern. Med..

[22] Turmo I.G. (2012). The Mediterranean diet: Consumption, cuisine and food habits. MediTERRA.

[23] García Cabrera S., Herrera Fernández N., Rodríguez Hernández C., Nissensohn M., Román-Viñas B., Serra-Majem L. (2015). KIDMED test; prevalence of low adherence to the mediterranean diet in children and young; a systematic review. Nutr. Hosp..

[24] Iaccarino Idelson P., Scalfi L., Valerio G. (2017). Adherence to the Mediterranean Diet in children and adolescents: A systematic review. Nutr. Metab. Cardiovasc. Dis. NMCD.

[25] Santomauro F., Lorini C., Tanini T., Indiani L., Lastrucci V., Comodo N., Bonaccorsi G. (2014). Adherence to Mediterranean diet in a sample of Tuscan adolescents. Nutrition.

[26] Manzano-Carrasco S., Felipe J.L., Sanchez-Sanchez J., Hernandez-Martin A., Gallardo L., Garcia-Unanue J. (2020). Weight Status, Adherence to the Mediterranean Diet, and Physical Fitness in Spanish Children and Adolescents: The Active Health Study. Nutrients.

[27] Aberg M.A., Aberg N., Brisman J., Sundberg R., Winkvist A., Torén K. (2009). Fish intake of Swedish male adolescents is a predictor of cognitive performance. Acta Paediatr..

[28] van der Wurff I.S., von Schacky C., Berge K., Zeegers M.P., Kirschner P.A., de Groot R.H. (2016). Association between Blood Omega-3 Index and Cognition in Typically Developing Dutch Adolescents. Nutrients.

[29] Vassiloudis I., Costarelli V., Yiannakouris N., Apostolopoulos K. (2011). Obesity, adherence to the Mediterranean diet and energy balance behaviours in relation to academic performance in primary school children. Int. J. Obes. Suppl..

[30] Costarelli V., Koretsi E., Georgitsogianni E. (2013). Health-related quality of life of Greek adolescents: The role of the Mediterranean diet. Qual. Life Res. Int. J. Qual. Life Asp. Treat. Care Rehabil..

[31] Romero-Robles M.A., Ccami-Bernal F., Ortiz-Benique Z.N., Pinto-Ruiz D.F., Benites-Zapata V.A., Casas Patiño D. (2022). Adherence to Mediterranean diet associated with health-related quality of life in children and adolescents: A systematic review. BMC Nutr..

[32] Calella P., Gallè F., Di Onofrio V., Cerullo G., Liguori G., Valerio G. (2022). Adherence to Mediterranean diet in athletes: A narrative review. Sport Sci. Health.

[33] D’Angelo S., Cusano P.J.S.S. (2020). Adherence to the Mediterranean diet in athletes. Sport Sci..

[34] Philippou E., Middleton N., Pistos C., Andreou E., Petrou M. (2017). The impact of nutrition education on nutrition knowledge and adherence to the Mediterranean Diet in adolescent competitive swimmers. J. Sci. Med. Sport.

[35] San Mauro Martín I., Garicano Vilar E., González Fernández M., Villacorta Pérez P., Megias Gamarra A., Miralles Rivera B., Figueroa Borque M., Andrés Sánchez N., Bonilla Navarro M.Á., Arranz Poza P. (2014). Hábitos alimentarios y psicológicos en personas que realizan ejercicio físico [Nutritional and psychological habits in people who practice exercise]. Nutr. Hosp..

[36] Peraita-Costa I., Llopis-Morales A., Marí-Bauset S., Marí-Sanchis A., Marí-Sanchis S., Morales-Suárez-Varela M. (2020). Burnout Syndrome Risk in Child and Adolescent Tennis Players and The Role of Adherence to the Mediterranean Diet. Int. J. Environ. Res. Public Health.

[37] Scannell N., Villani A., Mantzioris E., Swanepoel L. (2020). Understanding the Self-Perceived Barriers and Enablers toward Adopting a Mediterranean Diet in Australia: An Application of the Theory of Planned Behaviour Framework. Int. J. Environ. Res. Public Health.

[38] Hackman C.L., Knowlden A.P. (2014). Theory of reasoned action and theory of planned behavior-based dietary interventions in adolescents and young adults: A systematic review. Adolesc. Health Med. Ther..

[39] Ashrafi E., Mansourian M., Ebadi Fard Azar F., Rahideh S.T., Ezadi B., Osmani F. (2023). Investigation of factors related to healthy eating behavior based on the developed theory of planned behavior in adolescents. J. Educ. Health Promot..

[40] Patton-Lopez M.M., Manore M.M., Branscum A., Meng Y., Wong S.S. (2018). Changes in Sport Nutrition Knowledge, Attitudes/Beliefs and Behaviors Following a Two-Year Sport Nutrition Education and Life-Skills Intervention among High School Soccer Players. Nutrients.

[41] Bjørnarå H.B., Westergren T., Sejersted E., Torstveit M.K., Hansen B.H., Berntsen S., Bere E. (2021). Does organized sports participation in childhood and adolescence positively influence health? A review of reviews. Prev. Med. Rep..

[42] Riebl S.K., Estabrooks P.A., Dunsmore J.C., Savla J., Frisard M.I., Dietrich A.M., Peng Y., Zhang X., Davy B.M. (2015). A systematic literature review and meta-analysis: The Theory of Planned Behavior’s application to understand and predict nutrition-related behaviors in youth. Eat. Behav..

[43] Berg C., Jonsson I., Conner M. (2000). Understanding choice of milk and bread for breakfast among Swedish children aged 11–15 years: An application of the Theory of Planned Behaviour. Appetite.

[44] Morissette É., Laramée C., Drapeau V., Couture S., Valois P., Goulet C., Provencher V., Lamarche B. (2015). Determinants of restrictive dietary behaviors among female high school athletes. Health Behav. Policy Rev..

[45] Laramée C., Drapeau V., Valois P., Goulet C., Jacob R., Provencher V., Lamarche B. (2017). Evaluation of a Theory-Based Intervention Aimed at Reducing Intention to Use Restrictive Dietary Behaviors Among Adolescent Female Athletes. J. Nutr. Educ. Behav..

[46] Meng Y., Manore M.M., Schuna J.M., Patton-Lopez M.M., Branscum A., Wong S.S. (2018). Promoting Healthy Diet, Physical Activity, and Life-Skills in High School Athletes: Results from the WAVE Ripples for Change Childhood Obesity Prevention Two-Year Intervention. Nutrients.

[47] WHO Multicentre Growth Reference Study Group (2006). WHO Child Growth Standards based on length/height, weight and age. Acta Paediatr..

[48] Ajzen I. (2024). Constructing a TPB questionnaire: Conceptual and methodological considerations, 2002. Integrating the theory of planned behavior and the self-determination theory to promote Mediterranean diet adherence: A randomized controlled trial. Health Well-Being.

[49] Canova L., Manganelli A. (2016). Fruit and vegetables consumption as snacks among young people. The role of descriptive norm and habit in the theory of planned behavior. TPM-Test. Psychom. Methodol. Appl. Psychol..

[50] Taghipour A., Miri M.R., Sepahibaghan M., Vahedian-Shahroodi M., Lael-Monfared E., Gerayloo S. (2016). Prediction of Eating Behaviors Among High-School Students Based on the Constructs of Theory of Planned Behavior. Mod. Care J..

[51] Serra-Majem L., Ribas L., Ngo J., Ortega R.M., García A., Pérez-Rodrigo C., Aranceta J. (2004). Food, youth and the Mediterranean diet in Spain. Development of KIDMED, Mediterranean Diet Quality Index in children and adolescents. Public Health Nutr..

[52] Hayes A.F., Montoya A.K., Rockwood N.J. (2017). The analysis of mechanisms and their contingencies: PROCESS versus structural equation modeling. Australas. Mark. J..

[53] Preacher K.J., Hayes A.F. (2008). Asymptotic and resampling strategies for assessing and comparing indirect effects in multiple mediator models. Behav. Res. Methods.

[54] Muros J.J., Zabala M. (2018). Differences in Mediterranean Diet Adherence between Cyclists and Triathletes in a Sample of Spanish Athletes. Nutrients.

[55] Kostopoulou E., Tsekoura E., Fouzas S., Gkentzi D., Jelastopulu E., Varvarigou A. (2021). Association of lifestyle factors with a high prevalence of overweight and obesity in Greek children aged 10-16 years. Acta Paediatr..

[56] Makri R., Katsoulis M., Fotiou A., Kanavou E., Stavrou M., Richardson C., Kanellopoulou A., Orfanos P., Benetou V., Kokkevi A. (2022). Prevalence of Overweight and Obesity and Associated Diet-Related Behaviours and Habits in a Representative Sample of Adolescents in Greece. Children.

[57] Martinsen M., Bratland-Sanda S., Eriksson A.K., Sundgot-Borgen J. (2010). Dieting to win or to be thin? A study of dieting and disordered eating among adolescent elite athletes and non-athlete controls. Br. J. Sports Med..

[58] Natour N., Badrasawi M., Samuh M.H., Khodour R. (2020). Relationship Between Adherence to Mediterranean Diet with, Nutritional and Physical Fitness Status Among Young Athletes from Football Sports Academy- Palestine. Epidemiol Public Health.

[59] Alacid F., Vaquero-Cristóbal R., Sánchez-Pato A., Muyor J.M., López-Miñarro P.Á. (2014). Adhesión a la dieta mediterránea y relación con los parámetrosantropométricos de mujeres jóvenes kayakistas [Habit based consumptions in the mediterranean diet and the relationship with anthropometric parameters in young female kayakers]. Nutr. Hosp..

[60] Martínez-Rodríguez A., Sánchez-Sánchez J., Martínez-Olcina M., Vicente-Martínez M., Yáñez-Sepúlveda R., Cortés-Roco G., Vázquez-Diz J.A., Sánchez-Sáez J.A. (2022). Professional Male Beach Handball Players Performance Profile. Nutrients.

[61] Santana M.V., Mirón I.M., Vargas L.A., Bedoya J.L. (2019). Comparative analysis of adherence to the mediterranean diet among girls and adolescents who perform rhythmic gymnastics. Rev. Bras. De Med. Do Esporte.

[62] Sheeran P., Maki A., Montanaro E., Avishai-Yitshak A., Bryan A., Klein W.M., Miles E., Rothman A.J. (2016). The impact of changing attitudes, norms, and self-efficacy on health-related intentions and behavior: A meta-analysis. Health Psychol. Off. J. Div. Health Psychol..

[63] Hagger M.S., Chan D.K.C., Protogerou C., Chatzisarantis N.L.D. (2016). Using meta-analytic path analysis to test theoretical predictions in health behavior: An illustration based on meta-analyses of the theory of planned behavior. Prev. Med..

[64] McDermott M.S., Oliver M., Simnadis T., Beck E.J., Coltman T., Iverson D., Caputi P., Sharma R. (2015). The Theory of Planned Behaviour and dietary patterns: A systematic review and meta-analysis. Prev. Med..

